# Receptors Mediating Host-Microbiota Communication in the Metaorganism: The Invertebrate Perspective

**DOI:** 10.3389/fimmu.2020.01251

**Published:** 2020-06-16

**Authors:** Katja Dierking, Lucía Pita

**Affiliations:** ^1^Department of Evolutionary Ecology and Genetics, Zoological Institute, Christian-Albrechts-Universität zu Kiel, Kiel, Germany; ^2^RD3 Marine Symbioses, GEOMAR Helmholtz Centre for Ocean Research, Kiel, Germany

**Keywords:** innate immunity, pattern recognition receptors, holobiont, microbiome, invertebrate

## Abstract

Multicellular organisms live in close association with a plethora of microorganism, which have a profound effect on multiple host functions. As such, the microbiota and its host form an intimate functional entity, termed the metaorganism or holobiont. But how does the metaorganism communicate? Which receptors recognize microbial signals, mediate the effect of the microbiota on host physiology or regulate microbiota composition and homeostasis? In this review we provide an overview on the function of different receptor classes in animal host-microbiota communication. We put a special focus on invertebrate hosts, including both traditional invertebrate models such as *Drosophila melanogaster* and *Caenorhabditis elegans* and “non-model” invertebrates in microbiota research. Finally, we highlight the potential of invertebrate systems in studying mechanism of host-microbiota interactions.

## Introduction

A constantly and rapidly growing body of evidence supports the critical impact of the microbiota on multiple host functions, as diverse as digestion, development, metabolism, immune defenses, and behavior. In fact, almost every host process seems to be affected by the microbiota. As a consequence, hosts and their microbiota form an intimate functional entity, termed the “metaorganism” (1) or “holobiont” (2, 3). Metaorganism research aims at moving from correlation to causality, i.e., to understand how the microbiota shapes organism health and how microbiota and host activities emerge into metaorganism functions that also impact broader communities and ecosystems (4, 5). Moreover, the microbiota has become the target for novel therapies seeking to enhance health (6, 7), productivity (8), or even favor acclimation to new environmental conditions [e.g., (9)]. However, understanding the underlying mechanisms of host-microbiota interactions is key to translate metaorganism research into effective therapies and managing strategies.

Host-microbiota interactions are based on the exchange of information, which from the host point of view comes down to microbial signal—host receptor interactions. One group of host receptors that seem to play a crucial role in host-microbiota communication are the pattern recognition receptors (PRRs) of the innate immune system, such as Toll-like receptors (TLRs), NOD-like receptors (NLRs), and C-type lectin receptors (CTLRs) (10–12). PRRs recognize microbial molecules that are essential for microbes but absent in eukaryotic organisms, such as the cell surface molecules lipopolysaccharide (LPS), peptidoglycan (PGN), or flagellin (13, 14). As these so-called microbial-associated molecular patterns are produced by pathogenic as well as commensal bacteria, it was repeatedly suggested that PRRs, in addition to their crucial role in regulating defense responses to pathogens, may have evolved to communicate with commensal microbes [e.g., (10)]. Indeed, PRRs like TLRs and NLRs have been shown to mediate the effect of the microbiota on the immune system of the host [reviewed in (15, 16)]. Two other groups of host receptors that function in recognition of microbiota-derived signals are the G-protein coupled receptors (GPCRs) and peptidoglycan recognition proteins (PGRPs). GPCRs recognize bacteria-derived molecules, such as signal peptides or short chain fatty acids (17, 18).

In this review we summarize what is known about invertebrate host-microbe communication, with a particular focus on the role of the above-mentioned receptor classes, for which evidence of an involvement in host-microbiota interactions is available. For each receptor class, we will first give a brief overview and present examples of their function in mediating microbiota-host interactions in humans and mice. We will then review experimental evidence for a role of these receptors in microbiota-host interactions in invertebrates (Table 1). We focus not only on classical model organisms like the fruit fly *Drosophila melanogaster* or the nematode *Caenorhabditis elegans*, but also summarize evidence from diverse other taxonomic groups. We specify if evidence comes from genomic data, differential gene expression analysis or functional analysis. Finally, we will discuss the potential of invertebrate systems for the study of host-microbiota communication.

**Table 1:** Evidence of invertebrate PRR and GPCR function in host-microbiota communication.

	**Genomic Features^a^**	**Role in the metaorganism**	**References**
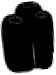 Porifera	TIR-Ig domain receptors Expansion of NLRs, SRs No PGRPs	SRCR up-regulated in symbiotic vs. aposymbiotic sponges	(19–24)
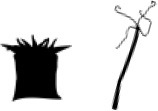 Cnidaria	TLRs in *Nematostella* but lack of *bona fide* TLR in *Hydra* and corals *Hydra* spp. lack *bona fide* NLRs; NLR expansion in *Nematostella* SRs expansion in corals	Bacterial colonization in *Hydra* mediated by *MyD88* Enhanced expression of SRs in symbiotic vs. aposymbiotic anemones. Impaired colonization if blocking SRs	(25–33)
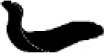 Annelida	TLR expansion in *Capitella teleta* Unclear function of PRRs		(34)
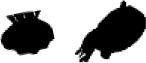 Mollusca	TLR expansion in certain species	PGRPs in symbiosis establishment in *Euprymna scolopes*	(35–39)
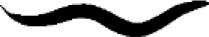 Nematoda	No NLRs or PGRPs Expansion of CTLRs and GPCRs	*C. elegans* TLR encoding gene *tol-1* for the protective effect of the *Enterococcus faecium*-derived secreted peptidoglycan hydrolase that enhances host tolerance to *Salmonella* infection CTLRs in microbiota recognition and aggregation in the cuticle of the marine nematode *Laxus oneistus*	(40–46)
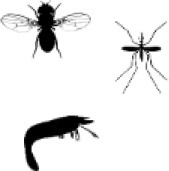 Arthropoda	Highly diverse phylum and, accordingly, genomic features depend on the group	TLRs no key role in symbiosis Mosquito CTLRs for facilitating microbiota persistence in the gut. Shrimp CTLR-mediated prevention of gut microbiota overgrowth PGRP and Imd pathway key in gut-microbiota interactions GPCRs: potential role in microbiota vs. pathogen distinction in *Drosophila*	(47–63)
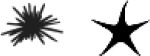 Echinodermata	Expansion of NLRs and SRs		(64–67)

*Silhouette images downloaded from PhyloPic or drawn by the Authors in Inkscape (no copyright)*.

a *According to specified references and/or Buckley and Rast (64). CTLR, C-type lectin receptors; GPCR, G-protein coupled receptor; Ig, immunoglobulin; NLR, NOD-like receptor; PGRP, peptidoglycan recognition protein; PRR, pattern recognition receptor; SR, scavenger receptor; SRCR, scavenger receptor cysteine-rich; TIR, Toll-Interleukin receptor; TLR, Toll-like receptor*.

## Toll-like Receptors (TLRs)

TLRs are transmembrane receptors with several extracellular leucine-rich repeat (LRR) motifs and an intracellular Toll/interleukin-1 receptor (TIR) domain. The extracellular LRR motifs of TLRs can bind a wide range of microbe-derived signals (e.g., LPS, flagellin, PGN, lipoteichoic acid), but also endogenous ligands derived from damaged cells such as the extracellular matrix molecules fibronectin and biglycan [reviewed in (68)]. TLR stimulation ultimately leads to the nuclear translocation of the transcription factors NF-κB or c-Jun and subsequently to the production of inflammatory cytokines or antimicrobial peptides (AMPs). In addition to NF-κB signaling, TLR receptors can activate mitogen-activated protein kinase (MAPK) and interferon regulatory factor signaling cascades [reviewed in: (69, 70)]. Extensive reviews on the evolution of TLRs, their ligands and downstream signaling cascade can be found in Brennan and Gilmore (71) and Nie et al. (72).

### Human and Mouse

The role of TLRs in microbial recognition is well-studied, particularly in the context of pathogens. But in 2004 the group of Ruslan Medzhitov proposed two distinct TLR functions—host defense against infection through recognition of pathogens and control of intestinal homeostasis through recognition of commensal bacteria: TLR-2-, TLR-4-, and MyD88-deficient mice showed increased susceptibility to intestinal injury induced by administration of dextran sulfate sodium (DSS) (73). Interestingly, germ-free mice were also highly susceptible to DSS-induced intestinal injury but could be rescued by administration of LPS. The authors concluded that the protection against intestinal injury occurred through recognition of commensal products by TLRs (73). This study was the first to describe a function of TLRs in the crosstalk between host innate immunity and the microbiota and thus in the control of intestinal homeostasis. Since then several studies have provided evidence of the involvement of TLRs in immunomodulation by the microbiota. For example, microbiota-derived polysaccharide A signals through TLR2 to suppress TH17 responses (74) and TLR 2 is required to sense outer membrane vesicle-associated polysaccharide from *Bacteroides fragilis* (75).

It thus seems that TLRs and also other PRRs are sentinels of microbial colonization by microbes in general, not only by pathogens. This makes sense as the microbial ligands of these receptors are not only produced by pathogens, but also commensal or beneficial bacteria. The challenge for the host is to detect microbial signals and interpret them in the appropriate context, preventing over-activation of defense responses and thus tolerating beneficial microbes while controlling overgrowth and responding appropriately to pathogens or opportunists. Vatanen et al. showed that the different members of the human gut microbiome present different LPS immunogenicity, as measured by TLR4 and NF-kB activation (76). D'Hennezel et al. showed that LPS of gut commensals of the order *Bacteroidales* silences TLR4 signaling and they proposed that this immunoinhibitory activity may sustain the tolerance of microbes in the gut (77). Decreased apical surface expression of TLRs and spatial segregation of host cells and commensal bacteria by mucus layers also prevent over-activation of TLR signaling (78, 79). The spatial segregation of microbiota and host epithelium depends on the regulatory feedback loop that senses bacterial colonizers via TLR-signaling and the activation of the expression of the antibacterial lectin RegIIIγ (80).

In addition, TLRs seem to play an important role in shaping and regulating the intestinal microbiota, as suggested by several studies analyzing the effect of TLR deficiency on microbial composition (81, 82). However, knock-out and wildtype mice used in these studies were separately maintained over multiple generations and a subsequent study comparing wild type and TLR-deficient littermate control mice, in which offspring resulted from crosses of mice heterozygous for each depletion identified no significant changes in the intestinal microbiota composition (83). The role of TLRs in regulating microbiota composition thus remains controversial.

Together, TLRs and TLR signaling play a crucial role in the recognition of both pathogenic and commensal bacteria and in the reciprocal interaction between the immune system and the intestinal microbiota in human and mice. It is unclear in how far TLRs directly mediate regulation of gut microbial composition, but it appears that balanced TLR signaling is most important to maintain intestinal homeostasis.

### Invertebrates

The first Toll receptor was discovered in *D. melanogaster*. Drosophila *Toll* is expressed in many tissues in a complex spatial and temporal pattern (84) and was initially identified as essential in *Drosophila* early embryonic development (85). It was subsequently found that in adult flies Toll signaling mediates defense responses against bacterial and fungal pathogens by regulating, among others, the expression of the antifungal peptide drosomycin in the fat body (47, 86). Only one Toll homolog, termed TOL-1, was identified in *C. elegans* but the worm lacks central proteins of the canonical TLR-signaling cascade such as NF-kB (48). TOL-1 is expressed in neurons and not required for *C. elegans* resistance to a number of pathogens, but for development (48) and also for development of chemosensory neurons that function in microbe sensing (87). *C. elegans tol-1* mutants are thus defective in pathogen avoidance behavior (87, 88). The first functional study of TLR signaling in a non-bilaterian animal demonstrated that recognition of bacteria and its contribution to defense is an ancestral function of TLRs (25). In the anthozoan *Nematostella vectensis* (phylum Cnidaria) a single TLR receptor could be identified (89) that is expressed in cnidocytes, stimulatedby flagellin *in vitro* and is involved in the recognition of the pathogen *Vibrio coralliilyticus* (26). In *Hydra* (phylum Cnidaria), however, conventional TLRs are absent; yet, they express a LRR domain protein that interacts with a TIR domain-containing protein and recognizes bacterial flagellin *in vitro* (90). In sponges (phylum Porifera), the function of TLR signaling is unknown. However, transcriptomic and genomic analyses of different sponge species from four different classes identified all essential genes involved in TLR signaling, but no conventional TLR (19–21, 91–93). Instead, sponges contain a receptor class with a TIR domain homolog of the TIR-domain of vertebrate TLRs, combined with extracellular immunoglobulin domains rather than LRR motifs. Components of the TLR pathway such as *MyD88* were activated in response to microbial signals in some sponge species (94, 95). The role of these Poriferan receptors and the TLR pathway in bacterial recognition thus remains to be probed (21).

While the function of TLRs in pathogen recognition and immune defense has been first demonstrated in an invertebrate, the role of TLRs in the communication between the microbiota and invertebrate hosts is less clearly defined. Intriguingly, The Toll pathway does not play a role in regulating gut homeostasis in *Drosophila*. Instead, flies rely on the immune deficiency (Imd) pathway, which is activated by peptidoglycan recognition proteins (PGRPs, see section below) for host-microbiota crosstalk (96). There are only two studies reporting experimental evidence of the involvement of invertebrate TLRs in mediating host-microbiota interactions, one in Hydra and one in *C. elegans*. Analysis of MyD88-deficient *Hydra* polyps revealed that TLR-signaling affects microbiome resilience after antibiotic disturbance (25). The only *C. elegans* TLR encoding gene *tol-1* was found to be required for the protective effect of the *Enterococcus faecium*-derived secreted peptidoglycan hydrolase SagA that enhances *C. elegans* tolerance to *Salmonella* infection (40). SagA remodels the peptidoglycan and generates muramyl-peptide fragments, which protect wildtype, but not *tol-1* mutant worms from *Salmonella* pathogenesis (40). However, it remains unclear how exactly *tol-1* is involved in SagA-mediated protection.

In summary, TLR signaling in invertebrates is functionally studied only in few model systems, yet recognition of bacteria seems to be an ancestral function of TLR signaling. Several studies in cnidarians, mollusks, and annelids have shown that bacterial recognition modulates the expression of genes encoding TLR pathway components [reviewed in (71)]. Further studies are needed to explore the potential function of TLR signaling in invertebrate host-microbe communication.

## NOD-like Receptors (NLRs)

Also known as nucleotide binding oligomerization domain (NOD)-like receptors, the standard nomenclature for the family has been designated “nucleotide-binding domain and leucine-rich repeat containing” receptors (NLRs), in order to emphasize the presence of these two conserved domains in *bona fide* NLRs (97): a nucleotide-binding domain denoted NACHT and a C-terminal LRR domain. The architecture of metazoan NLRs usually includes a third N-terminal domain, mainly a Death domain, a CARD domain or a Pyrin domain [reviewed in (97)]. NLRs exemplify how investigating different animal groups broadens our knowledge on the evolution of the immune system. Because *D. melanogaster* and *C. elegans* lack NLRs, it was long thought this family had its origin in teleost fish. The availability of genomes and transcriptomes from non-model invertebrates, such as the publication of the sea urchin *Strongylocentrotus purpuratus* genome (98) and later the sponge *Amphimedon queenslandica* genome (20), revealed the ancient origin of animal NLRs (22). The evolutionary trajectory of animal NLRs is complex and diverse, with remarkable losses and expansions. Expansions of NLRs occurred in the sponge *A. queenslandica* (135 genes), the sea urchin *S. purpuratus* (203 genes) (22), the coral *Acropora digitifera* (66 genes, (99)), the lancelet *Branchiostoma floridae* [92 genes, as calculated by (64)], and in the zebra fish *Danio rerio* [>250 genes, (100)]. In contrast, the cnidarian *Hydra magnipapillata*, the ctenophore *Mnemiopsis* sp., the nematode *C. elegans*, the urochordate *Okopleura dioica*, and various arthropods lack *bona fide* NLRs (22). Some of these animals, such as *Hydra*, harbor instead a diverse repertoire of proteins containing a nucleotide binding domain that could potentially have a similar role as *bona fide* NLRs (27).

### Human and Mouse

Most of our knowledge about NLR signaling pathways and function in animal-microbe interactions comes from research on mice and human cell lines [reviewed in (101)]. NLRs recognize microbes through the C-terminal LRR domain by direct binding a diverse range of ligands that include LPS, PGN, and small bacterial peptides [e.g., (102, 103)]. Microbial recognition can also happened indirectly; for example, in HEK293 cells, the exposure to *Salmonella*-derived proteins activated the small Rho-GTPases such as RAC1 and the active GTP-bound state induced NOD1-dependent signaling (104). The N-terminal domain engages in protein-protein interactions and triggers downstream signaling cascades. Mammalian NLRs, such as NOD1 and NOD2, mainly activate NF-κB, but they can also induce MAPK signaling cascades (e.g. JNKs, ERKs, p38) [reviewed in: (101, 105)]. Other NLRs form multimeric complexes known as “inflammasomes” that consists of one or several NLRs, an adapter protein containing a CARD domain, and a caspase as effector [reviewed in (106)]. NLR activation and subsequent signaling result in regulation of reactive oxygen species formation, secretion of cytokines, production of AMPs, as well as apoptosis [reviewed in: (105, 107)].

NLRs have received great attention in the context of human-gut microbiota interaction because mutations in *NLR* genes were repeatedly associated with chronic inflammatory diseases (e.g., *Nod2* mutation as a risk factor for Crohn's disease) [reviewed in (101, 108)]. Impairment of *Nod2* and *Nlrp 6-*inflammasome genes in a mouse model correlated with dysbiosis, suggesting that these receptors could regulate gut microbiota composition [e.g., (109, 110)]. However, other studies could not detect differences in microbiota composition between *Nod1, Nod2*, or *Nlrp6* deficient mice and wild type mice, when controlling for mouse breeding and housing effects [e.g., (111–113)].

In a different approach, Schieber et al. (114) showed that the commensal gut bacterium *E. coli* O21:H^+^ act via the *Nlrc4* inflammasome to promote disease tolerance as defense during infection and tissue inflammation. Upon infection by *Burkholderia thailandesis* (a model for pneumonic infection which provokes wasting of skeletal muscle but does not compromise intestinal barrier), the commensal *E. coli* O21:H^+^ translocates into the white adipose tissue and activates the *Nlrc4* inflammasome to induce sustained insulin-like growth factor 1 signaling in the skeletal muscle, resulting in prevention of muscle loss (114). Thus, the location of the microbial signal is also relevant for determining host response. Recently, Kim et al. (115) showed that the probiotic effect of *E. faecium* against *Clostridium difficile* pathogenesis in mice depends on activation of NOD2. NOD2 is activated by small muropeptides that are generated by the *E. faecium*-derived peptidoglycan hydrolase SagA. These studies provide evidence of key interactions between NLRs and commensal bacteria and the consequences for host health.

Thus, mouse NLRs detect microbial signals derived from commensal and pathogenic bacteria, but the direct link between these NLRs and the gut microbiota composition remains highly debated and needs to be resolved by future studies.

### Invertebrates

To what extent do the NLR functions described in mammals apply to invertebrates? The expansion of the NLR family and genetic diversity of the recognition domain (i.e., high polymorphism of C-terminal LRRs) is considered an indication for specific recognition of diverse microbial ligands (116). However, only a handful of studies provide evidence of a potential role of NLRs in invertebrate host-microbiota interactions, and all are based on gene expression analysis. Sponges expressed a high diversity of NLRs and, recently, the enhanced expression of NLRs in response to PGN and LPS was reported (21). In the medicinal leech *Hirudo medicinalis* (phylum Annelida), a LRR-domain containing protein with sequence similarity to vertebrate NLRs was identified and its expression in nerve chords was enhanced upon exposure to heat killed *Micrococcus nishinomiyaensis*, as well as to *E. coli* LPS, lipoteichoic acid, and muramyl dipeptide (34). Finally, a non-conventional *Hydra* NLR protein, mainly expressed in the endoderm of this animal, responded to LPS and flagellin stimulation and recruited an effector caspase in a heterologous expression system (27). Thus, the expansion of NLRs in certain groups and these first results suggest that invertebrate NLRs are good candidates to mediate host-microbiota crosstalk.

## C-type Lectin Receptors (CTLRs)

The C-type lectin-like domain family contains secreted as well as transmembrane proteins that are highly diverse regarding their overall domain architecture, but all share primary and secondary structural homology in their carbohydrate recognition domain [reviewed in: (117, 118)]. The first described members of this family indeed bound carbohydrates in a calcium-dependent (C-type) manner, and were thus *bona fide* lectins. However, the carbohydrate recognition domain was subsequently also identified in proteins that did not bind carbohydrates, but other ligands such as proteins and lipids, and also did not require calcium for binding. The term C-type lectin-like domain (CTLD) was thus introduced to reflect the structural similarity to the carbohydrate recognition domain of prototype C-type lectins without implying common function (119). CTLD genes are abundant in metazoan genomes and constitute highly diverse and expanded gene families. The human genome encodes 100 CTLD genes, 132 CTLD genes are encoded in the mice genome, 56 CTLD genes in *D. melanogaster*, 283 CTLD genes in *C. elegans*, 67 CTLD genes in *Nematostella vectensis*, and 2 CTLD genes in the sponge *A. queenslandica* [reviewed in (49)].

### Human and Mouse

CTLD proteins perform multiple functions in human and mouse immune defense and are commonly called C-type lectin receptors (CTLRs). CTLRs function as PRRs that bind glycans, such as mannose, fucose, and N-acetylgalactosamine residues on the surface of pathogens, including bacteria, fungi, viruses, and parasites, and regulate innate and adaptive immune responses. Other CTLRs bind endogenous ligands (self-antigens) and thus play an important role in immune homeostasis [for a review of CTLR functions in the human/mouse immune system see (120, 121)]. The high diversity of glycosylated bacterial proteins or lipids attached to the cell surface or to secreted molecules constitute ideal ligands to establish specific interactions with the host (122).

CTLRs are crucial for the recognition of bacterial glycoconjugates in the context of pathogen infection and there is some evidence for CTLR ligands from commensal bacteria. For example, the beneficial human gut bacterium *Lactobacillus acidophilus* produces the glycosylated surface layer A protein, which modulates human dendritic cell and T-cell responses via the interaction with the CTLR dendritic cell-specific intercellular adhesion molecule-3-grabbing non-integrin (DC-SIGN) (123). Similarly, *Lactobacillus rhamnosus* produces a glycosylated adhesive heterotrimeric pili, which interacts with DC-SIGN resulting in modulation of the cytokine response of human dendritic cells (124). These two *Lactobacillus* glycoproteins are the as yet only identified CTLR ligands from commensal bacteria. Furthermore, two studies provide evidence of an interaction between CTLRs and commensal bacteria and its impact on gut homeostasis in experimental murine colitis models. The closest murine homolog of DC-SIGN, the specific intracellular adhesion molecule-3 grabbing non-integrin homolog-related 3 (SIGNR3), interacted with *L. acidophilus* surface layer protein A contributing to the maintenance of healthy gastrointestinal microbiota, protection of the gut mucosal barrier function, and mitigation of colitis (125). Hütter and colleagues showed that the two CTLRs, macrophage C-type lectin (MCL) and dendritic cell immunoreceptor (DCIR), bind commensal intestinal bacteria *in vitro* (126). However, MCL^−/−^ and DCIR^−/−^ knock-out mice showed only a slight increase in inflammation in a DSS murine colitis model and the role of MCL and DCIR in regulating gut homeostasis thus remains unclear. Interestingly, recognition of commensal fungi by CTLRs also seems to support intestinal immune homeostasis. Dectin-1, a major PRR in antifungal immunity, and SIGNR3 regulated the host response to commensal fungi and influenced immune homeostasis in a DSS murine colitis model (127, 128). Moreover, a polymorphism of the Dectin-1/CLEC7A gene in humans was associated with severe ulcerative colitis (127). Together, these studies demonstrate an emerging role of vertebrate CTLD proteins in microbiota-host interactions, including not only bacteria but also fungi.

### Invertebrates

In invertebrates, CTLD genes contribute to immune responses in a variety of different taxa, including insects, crustaceans, and nematodes [reviewed in (49)]. Insect and crustacean CTLD proteins are involved in cellular immune responses, such as hemocyte nodule formation, encapsulation, melanization, and activation of phagocytosis, and in the direct elimination of pathogens by exhibiting antimicrobial activity [reviewed in (49)]. In *C. elegans*, CTLD genes are mainly expressed in the intestine and can mediate both physiological, as well as behavioral immune responses (41). Invertebrate CTLD proteins were thus suggested to function as PRRs. We however know almost nothing about the downstream signaling pathways that are activated by invertebrate CTLD proteins. The one exception is a study on the CTLD proteins FcLec4 from the kuruma shrimp *Marsupenaeus japonicas*, which binds β-integrin in the membrane of hemocytes to promote phagocytosis (50).

**Figure 1:**
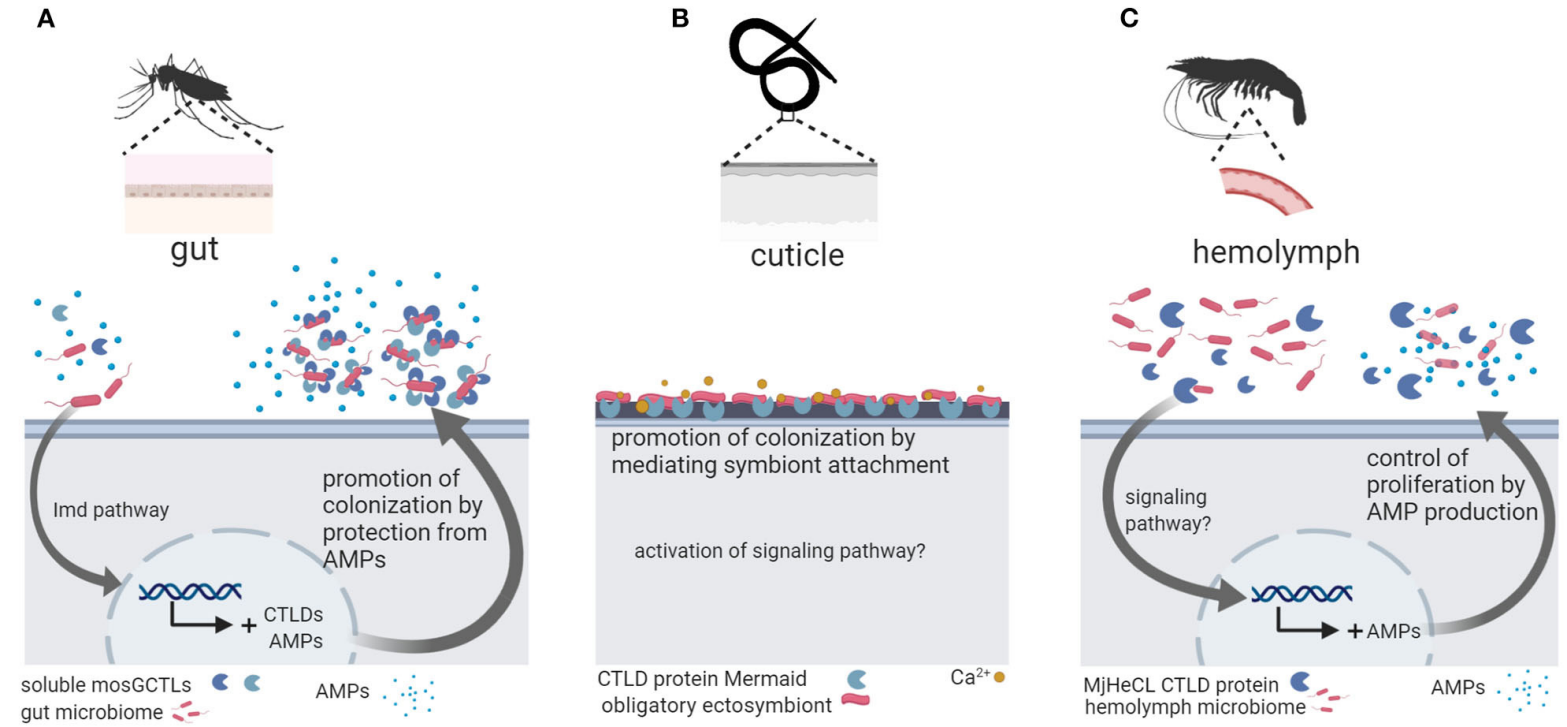
Invertebrate C-type lectin-like domain (CTLD) proteins function in host colonization by the microbiota. **(A)** In the mosquito *Aedes aegypti*, gut microbiota bacteria are coated by soluble mosCTLs and, in this way, protected from antimicrobial peptides (AMPs) secreted by the host, promoting colonization and persistence in the gut (51). **(B)** In the marine nematode *Laxus oneistus*, the calcium-dependent CTLD protein Mermaid mediates the agglutination and attachment of the obligatory ectosymbiont, a sulfur-oxidizing bacterium that forms a monolayer in the cuticle. If symbiont attachment via Mermaid leads to activation of intracellular signaling remains unknown. **(C)** In the kuruma shrimp *Marsupenaeus japonicas*, the CTLD protein MjHeCL binds microbiota bacteria in the hemolymph and activates AMP production to prevent bacterial overgrowth. Figure created with Biorender.com. Silhouette image for *L. oneistus* was downloaded from PhyloPic (no copyright).

Three studies linked CTLD protein function to host colonization by microbiota bacteria in invertebrates (Figure 1). In the mosquito *Aedes aegypti* the CTLD proteins *mosGCTL-29* and *mosGCTL-32* seem to facilitate colonization and persistence of microbiota bacteria in the gut: *mosGCTL-29* and *mosGCTL-32* bind *E. coli* cells and protect *E. coli* and microbiota bacteria against AMP activity by coating the bacterial surface. Silencing the expression of *mosGCTL-29* and *mosGCTL-32* by RNAi or blocking the CTLD proteins by feeding mosquitos mosGCTL antisera, reduced bacterial colonization (51). In contrast, the CTLD protein MjHeCL from the kuruma shrimp *M. japonicas* controls the hemolymph microbiota by inhibiting bacterial proliferation (52). Silencing the expression of MjHeCL by RNAi increased proliferation of the hemolymph microbiota. MjHeCL was shown to bind to several hemolymph microbiota isolates and to be required for expression of certain AMPs in *M. japonicas* hemocytes. It was thus suggested that MjHeCL plays a role in restricting the growth of the hemolymph microbiota by regulating AMP expression (52). Finally, a CTLD protein mediates symbiont attachment in the marine nematode *Laxus oneistus*. The *L. oneistus* cuticle is covered by a monolayer of a single phylotype of sulfur-oxidizing bacteria. The mannose-specific CTLD protein Mermaid, which is similar to human DC-SIGN, is secreted onto the cuticle and mediates symbiont aggregation and attachment to the worm (42). Moreover, different Mermaid isoforms serve to discriminate different bacterial symbionts and were thus suggested to be involved in the specific recruitment of symbionts (43).

Taken together, future investigations in other animal groups will help understanding the role of invertebrate CTLD proteins in host-microbiota interactions.

## Peptidoglycan Recognition Proteins (PGRPs)

PGRPs (also known as PGLYRPs in mammals) are a family of homologous receptors characterized by the presence of at least one PGRP domain with structure and sequence similarity to bacteriophage type 2 amidases [reviewed in (53)]. PGRPs are secreted, transmembrane, or intracellular proteins that bind and often also hydrolyze PGN. However, certain PGRPs can also bind other molecules such as LPS and lipoteichoic acid [reviewed in (53)]. PGRPs are present in many animals groups, from insects to mammals; yet absent from the genomes of representatives for other animal phyla such as the sponge *A. queenslandica*, the cnidarian *N. vectensis*, the nematode *C. elegans*, or the crustacean *Daphnia pulex* [reviewed in (64)]. In vertebrates, the suite of PGRPs is rather small (e.g., 4 PGRPs in mammals and in zebrafish). In zebrafish, PGRPs are expressed in skin, gills, liver, intestine, and pancreas (129) and have been implicated in immune defenses [reviewed in (53)]. In mammals, all 4 PGRPs (PGLYRP1-PGLYRP4) recognize PGN [reviewed in (53)], but may also recognize other ligands, including LPS [reviewed in (130)].

### Human and Mouse

Mammalian PGRPs have antibacterial activity and assist in macrophage activation during immune responses [reviewed in (130)]. They are expressed in different tissues, including the gastrointestinal tract [reviewed in (131)]. Saha et al. (132) investigated the role of PGRPs in the gut by analyzing DSS-induced colitis in mice that were deficient for individual *pglyrp* genes. All PGRP deficient mice were more susceptible to colitis and showed significant changes in gut microbiota composition (as bacterial abundances quantified by qPCR). Interestingly, deficiency in each *pglyrp* induced different changes, suggesting specific functions of these genes. When transferred into germ-free mice, stools from *pglyrp*-deficient mice produced higher inflammatory activity and increased sensitivity to colitis in the receiving mice than stools from wild type mice. Therefore, PGRPs might play a protective role in the gut by preventing inflammation upon damage, possibly, by directly regulating gut microbiota composition.

### Invertebrates

**Figure 2:**
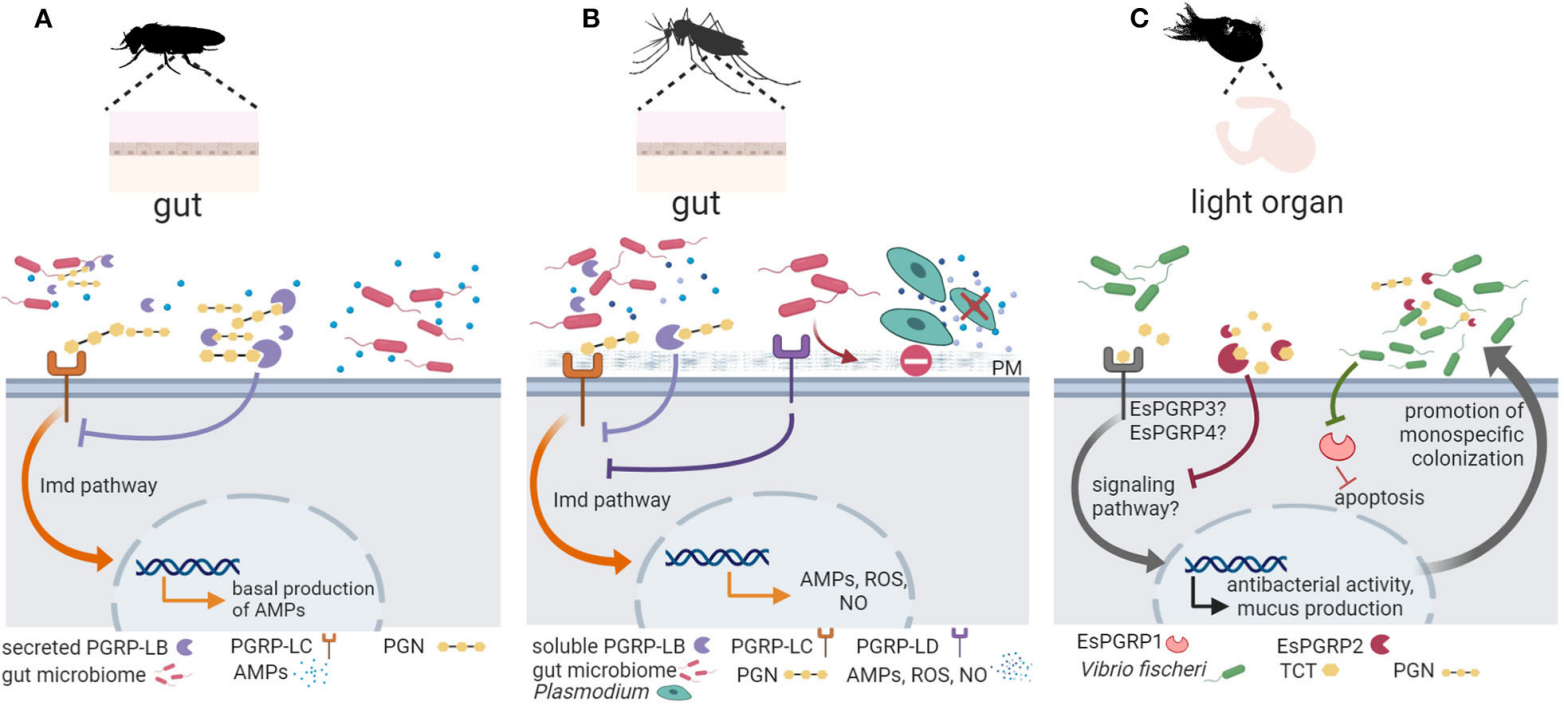
Invertebrate PGRP function in symbiont colonization and symbiosis homeostasis. **(A)** In the fruit fly *Drosophila melanogaster*, peptidoglycan recognition proteins (PGRPs) are important for keeping the balance between activating an immune response to pathogenic bacteria and preserving the beneficial microbiota [reviewed in (57)]. Peptidoglycan (PGN) derived from gut microbiota activates basal antimicrobial peptide (AMP) production via the PGRP receptor PGRP-LC (shown in orange) and the immune deficiency (Imd) signaling pathway. Secreted PGRP-LB (shown in purple) hydrolyzes excessive PGN downregulating antimicrobial defenses to promote host tolerance toward the microbiota (58). **(B)** In mosquitos, PGRPs mediate gut homeostasis and symbiosis. Similar to its function in *D. melanogaster*, PGRP-LC (shown in orange) detects microbiota derived PGN and activates the Imd pathway to promote immune effectors (AMPs, reactive oxygen species (ROS), nitric oxide (NO), whereas a soluble PGRP (PGRP-LB) (shown in purple) hydrolyzes excessive PGN to promote tolerance [see (53) and references therein]. In addition, the receptor PGRP-LD (shown in dark purple) also protects the gut microbiota by dampening immune activities, and this action is key to maintaining the integrity of the peritrophic matrix (PM), which acts as barrier against parasitic infection by *Plasmodium* (61). **(C)** In the symbiosis between the Hawaiian bobtail squid *Euprymna scolopes* and the bioluminescent bacterium *Vibrio fischeri*, the symbiont PGN-derived tracheal cytotoxin (TCT) activates mucus production to promote colonization. As symbiont colonization progresses, EsPGRP1 is silenced to induce apoptosis and rearrangement of the host light organ. The amidase activity of EsPGRP2 detoxifies TCT to promote symbiont tolerance. Figure created with Biorender.com. Silhouette image for *Drosophila* was downloaded from PhyloPic (no copyright).

In insects, PGRPs play a key role in pathogen defense [reviewed in (53)]. In the sea star *Asterias rubens*, two secreted PGRPs have been characterized, PGRP-S1a, present in the coelomic plasma, and PGRP-S2a, expressed in phagocytes. PGRP-S2a opsonized *Micrococcus luteus*, increasing their phagocytosis (65). Importantly, accumulating experimental evidence supports a crucial role of PGRPs in fly and mosquito host-microbiota crosstalk and in the symbiosis between the Hawaiian bobtail squid *Euprymna scolopes* and the bioluminescent bacterium *Vibrio fischeri* (Figure 2).

In the fly, recognition of PGN through PGRPs activates the Imd pathway, which is in addition to the Toll pathway the major AMP-regulating signaling pathway in *Drosophila* (54, 55). While the Toll pathway only regulates AMP expression in the body cavity, the Imd pathway controls AMP expression in the body cavity and in the gut. The expression of AMPs in the gut is strongly reduced in axenic flies, which indicates that the gut microbiota activates the Imd pathway, leading to a basal expression of AMPs in the presence of commensal bacteria (56, 133). *D. melanogaster* has 13 PGPR genes that encode more than 20 transmembrane, cytoplasmic or soluble proteins, which act in different immune tissues such as the fat body, hemocytes, and barrier epithelia in the gut and trachea (53). While some PGRPs function as recognition proteins and activate antimicrobial responses, other PGRPs function as inhibitory receptors or PGN-hydrolyzing amidases that downregulate antimicrobial defenses. *Drosophila* PGRPs thus seem to play an important role in keeping the balance between activating an immune response to pathogenic bacteria and preserving the beneficial microbiota [reviewed in (57)]. For example, secreted and cytosolic isoforms of the PGN-cleaving amidase PGRP-LB control the level of extracellular PGN in the gut lumen and of intracellular PGN inside enterocytes, respectively, to prevent constitutive activation of NF-κB in response to microbiota bacteria (58). Moreover, the extracellular receptor PGRP-SD, which is important for the detection of bacterial pathogens in *Drosophila* (59), also affect microbiota composition: The intestinal microbiota of PRGP-SD knock-out flies showed an increased abundance of *Lactobacillus plantarum*. The excessive proliferation of *L. plantarum* derived in excessive levels of metabolite lactic acid and resulted in the generation of reactive oxygen species, which in turn promoted intestinal damage and increased proliferation of intestinal stem cells, and dysplasia (60).

In mosquitos, there is strong support for the role of PGRPs in regulating gut homeostasis and promoting symbiosis. Song et al. (61) showed that knockdown of *Aedes stephensi* PGRP-LD activated immune responses and changed the abundance and spatial distribution of the gut microbiota. Reduction of the microbiota in PGRP-LD knock down mosquitoes and in antibiotic-treated mosquitos compromised the peritrophic matrix, which represents the physical barrier of mosquito midgut epithelium and its luminal contents (61). Thus, PGRP-LD dampens immune responses to protect gut microbiota, which in turn maintains the structural integrity of the peritrophic matrix. In the mosquito *Aedes aegypti*, symbiosis with *Wolbachia* activates PGRP-LE, which is a receptor of the Imd pathway (134). In independent experiments using genetic tools to silence PGRP-LE or components and regulators of Imd and Toll pathways, the authors provide strong evidence of the role of PGRP-LE and downstream signaling in controlling symbiont load in *A. aegypti* (134).

In the Hawaiian bobtail squid *Euprymna scolopes*, recognition of LPS, PGN, and PGN derivate tracheal cytotoxin (TCT) is essential for the highly specific colonization of the *E. scolopes* light organ by *Vibrio fischeri* [reviewed in (14)]. PGN triggers mucus production, which facilitates *V. fischeri* aggregation, and later LPS and TCT induce apoptosis and epithelium regression for the formation of the light organ. Ligand assays are missing, but PGRP expression profiles agree with their role in mediating the establishment of the symbiosis in the squid juvenile. For example, the *E. scolopes* PGRP *EsPGRP1* is expressed in the nucleus of epithelial cells but TCT alters its localization during the inducement of light organ morphogenesis (35). This effect disappears when the host encounters a *V. fischeri* mutant that is defective in the release of TCT (35). Another *E. scolopes* PGRP, EsPGRP2, degrades TCT via amidase activity, reducing its toxicity (36) and, thus, authors suggests that EsPGRP2 may help dampen the response to this powerful toxin during periods when symbiont concentrations are high and promote tolerance (36). EsPGRP5 is also predicted to present amidase activity and is highly expressed in the hemocytes (37), which are *E. scolopes* immune cells. *EsPGRP5* gene expression is altered in hemocytes from symbiotic vs. aposymbiotic hosts (37).

To summarize, PGRPs play a vital role in maintaining intestinal homeostasis in insects and in establishing the squid-*Vibrio* symbiosis, two distantly-related animal groups. The function of PGRPs in host-microbiota interactions in other invertebrates remains to be investigated.

## Scavenger Receptors (SRs)

SRs were first characterized based on their capacity to bind altered low-density lipoproteins (135). Since then, additional ligands have been identified, such as LPS, β-glucan, maleylated bovine serum albumin, and viruses [reviewed in (136)]. Despite similar ligand affinities, SRs comprise both secreted and transmembrane proteins that are structurally very heterogeneous. The high complexity of protein domain architectures reveals the lack of (or little) homology within this superfamily [reviewed in (136)]. Some of the domains reported in SRs include: the scavenger receptor cysteine-rich (SRCR) domains (class A and I), collagen (class A), CD36 (class B), or the epidermal growth factor (EGF) and EGF-like domains (class F and H). The exact binding capacity and downstream signaling vary between different SRs.

### Human and Mouse

Our understanding of the functions and signaling mediated by SRs relies mainly on results from mammals, where they play a role in immunity (e.g., inducing bacterial clearance) but also in homeostasis by regulating lipid transport [reviewed in (136)]. However, they have been little studied in the context of host-microbiota crosstalk. CD36 domain-containing SRs are found in all animals, from sponges to humans (28, 137, 138). They conform the SR-B class and probably the most studied one is the vertebrate receptors CD36 [reviewed in (136)]. CD36 binds diacyl fatty acids of microbial cells, acting as a PRR (139, 140). Moreover, CD36 mediates the recognition of apoptotic cells via the detection of modified lipids and thrombospondin-1 and is also involved in lipid transport (141). The signaling pathways activated by CD36 vary depending on the ligand. For example, binding thrompospondin-1 yields actin rearrangement and pro-apoptotic signals through MAPKs such as p38 or JNK [reviewed in (136)], whereas bacteria (139) and endogenous modified lipoproteins (142) induce inflammatory responses. In mice, CD36 function in host-microbiota crosstalk is mainly indirect and relies on its role in lipid metabolism, as the microbiota influences host lipid content and these changes affect *CD36* gene expression levels [e.g., (143, 144)].

### Invertebrates

In invertebrates, CD36 domain-containing SRs seem to be involved in the response to bacterial challenge in diverse animal groups. For example, the SR-B family member MjSR-B1 in the kuruma shrimp *Marsupenaue japonicus* is mainly expressed in hemocytes, hepatopancreas, and heart (62). *Mj*SR-B1 knockdown impairs bacteria agglutination, phagocytosis and expression of antimicrobial peptides upon exposure to the pathogens *Vibrio anguillarum* and *Staphyloccoccus aureus*, whereas overexpression of *Mj*SR-B1 has the opposite effect (62). In the octopus *Octopus ocellatus, Oo*SR-B localizes to the surface of hemocytes and stimulates phagocytosis upon exposure to *V. anguillarum* and to *Micrococcus luteus* (38). Knockdown of *Oo*SR-B impaired the transcription of TLRs and downstream components of the cascade (i.e., MyD88 and TRAF6), suggesting that the interaction of *Oo*SR-B and TLRs is key in the response to bacteria (38). A CD36-like gene is strongly upregulated in symbiotic *vs*. aposymbiotic (i.e., dinoflagellate-free) sea anemones *Aiptasia* sp. and *Anthopleura elegantissima* (phylum Cnidaria) (29, 30); however, exact role cnidarian CD36 domain-containing SRs play in cnidarian-dinoflagellate symbiosis is unknown.

In invertebrates, the group of SRCRs has received special attention due to their high diversity in the genome of several animal groups. In contrast to mammals (145), invertebrate SRCR protein architectures are highly diverse: SRCR domains are reported as single domains, arranged in tandem and/or in combination with other conserved domains such as immunoglobulin domains, collagen, low density lipoprotein receptor, and fibronectin type III [e.g., (146–148)]. In terms of total number of SRCR domain and SRCR genes, some invertebrates showed similar or even lower diversification than mammals (e.g., 3 SRCR domains in *C. elegans*, 22 SRCR domains in *Ciona intestinalis*) (64). However, sea urchins, sponges and in less order of magnitude, lancelets (amphioxus) showed significant expansions of the SRCR gene family, with hundreds of genes reported in their genomes (64).

A handful of studies have explored the function of invertebrate SRCRs in immunity, mainly through gene expression analyses. In sponges, one SRCR gene was up-regulated in the Mediterranean species *Aplysina aerophoba* in response to LPS and PGN (21). Also, a diverse array of SRCRs is activated in juveniles of the sponge *Amphimedon queenslandica* when exposed to their own microbiota as well as to seawater bacteria (95). Interestingly, the expression of SRCR-containing genes is enriched in the choanocytes of adult *A. queenslandica*, which are phagocytic cells that are in direct contact with the external environment (149). In adults of the purple sea urchin *S. purpuratus* SRCRs are specifically expressed in coelomocytes, the sea urchin immune cells (150). The expression profiles were individual specific and fluctuated over time (e.g., their expression levels can fluctuate up to 10-fold in 1 week) (147). In *S. purpuratus* larvae, the SRCR gene *srcr142* was expressed in pigment cells, phagocytic cells and also in the amoeboid cells, which are the specialized immune cells in the larvae (66). Moreover, *srcr142* expression was enhanced upon exposure of larvae to the seawater bacterium *Vibrio diazotrophicus* (66). In the sea star *Asterina pectinifera*, a secreted SRCR protein (*Ap*SRCR1) is highly expressed in coelomocytes and mediates bacterial binding and clearance (67). In several coral species, the *dmbt1* gene, annotated based on protein sequence similarity, is differentially-expressed in response to LPS stimulation (31), during disease (151), and upon exposure to *Vibrio* bacterium (*Vibrio owensii* and *V. diazotrophicus*) (32). The scallop SRCR-containing gene *CfSR* was up-regulated upon stimulation with different microbial-associated molecular patterns (i.e., LPS, PGN, ß-glucan) and the protein was expressed at the surface of hemocytes (39). In this case, binding assays on the purified *Cf* SR protein showed its affinity for acetylated low density lipoprotein, dextran sulfate, LPS, PGN, zymosan, and mannan (39), which confirmed its function as PRR.

An additional study points to the potential role of SRCRs in regulating host colonization by the microbiota in sponges. Steindler et al. (23) identified a *SRCR*-containing gene as potential mediator in the establishment of the intracellular cyanobacterial symbiont in the Mediterranean sponge *Petrosia ficiformes*. Specimens of *P. ficiformis* become naturally aposymbiotic (loss of cyanobacteria) when growing in dark caves. In comparison to aposymbiotic specimens, symbiotic sponges (harboring cyanobacteria) living at a short distance in illuminated areas showed elevated expression of a gene containing a SRCR domain (23). The alignment of the predicted protein revealed similarity with the SRCR-containing protein derived from other sponge species, *Geodia cydonium*, as well as the sea urchin *S. purpuratus* and the zebrafish *Danio rerio*. However, further studies are necessary to unequivocally confirm the role of SRCR in symbiosis establishment in sponges.

Interestingly, Neubauer et al. (28) impaired the ability of the sea anemone *Aiptasia pallida* to acquire the endosymbiotic dinoflagellate *Symbiodium* by pre-incubating aposymbiotic anemones with the SR ligand fucoidan, which blocks the binding sites of SRs. Although the class of the blocked SR(s) was not identified, the experiment supports the role of SRs in the establishment of anemone-dinoflagellate symbiosis (28).

In summary, the great diversity of scavenger receptors and their role in invertebrate response to microorganisms, with preliminary evidence of a role in symbiosis establishment, makes this PRR group an interesting target for future studies on host-microbiota crosstalk.

## G Protein-coupled Receptors (GPCRs)

GPCRs are central for the perception of external stimuli and the transduction of the signal to the cellular cytoplasm and thus vital for the connection of the cell and organism to its environment. The family of GPCRs represents the largest receptor family in animals (152). The human genome contains over 800 GPCR-encoding genes, the mice genome comprises over 1,300 GPCRs and zebrafish genome includes over 700 GPCRs (153). In invertebrate genomes, 116 GPCRs were found in *D. melanogaster* (154), over 1,000 GPCRs in *C. elegans* (155), and 220 GPCRs in the sponge *Amphimedon queenslandica* (156). All GPCRs share a common structure and the general mechanism of activation [reviewed in (157)]: They are characterized by a conserved signature motif consisting of seven transmembrane (7TM) spanning helix domains. The extracellular N-terminal domain or the extracellular loops joining the 7TM domains mediate receptor activation through ligand binding, which causes conformational changes in the 7TM domain and in turn activates the cytoplasmic C-terminal domain. The activated C-terminal domain starts the intracellular signaling cascades typically through coupling to heterotrimeric guanine nucleotide-binding regulatory proteins (G proteins), but also through G protein-independent pathways mainly via G protein-coupled receptor kinases (GRKs) and arrestin [reviewed in: (158, 159)]. GPCR ligands are as diverse as photons, Ca^2+^, proteins, hormones, drugs, odorants, and small molecules such as amino acid residues, nucleotides, and peptides. GPCRs are thus involved in a multitude of different physiological functions, such as development, reproduction, metabolism, neuronal function, taste, smell, cell adhesion, immune function, and the regulation of feeding (160, 161).

### Human and Mouse

In vertebrates, GPCRs play an important role in the crosstalk between microbes and the host. In vertebrate immunity GPCRs function next to TLRs and NLRs in the recognition of microbial-associated molecular patterns [reviewed in (162)]. Cells of the innate and adaptive immune system abundantly express GPCRs, which regulate the immune response and cell migration by sensing both endogenous signals (e.g., chemokines and other inflammatory factors such as activated complement) and bacterial-derived signals [e.g., formyl peptides or short chain carboxylic acids (17, 18)]. GPCRs bind products of bacterial fermentation, in particular the short-chain fatty acids (SCFAs), induce migration and activate innate immune responses in human and mouse leukocytes [reviewed in (163)]. Besides, these GPCRs mediate the activation of AMP production by bacterial SCFAs in intestinal epithelial cells (164). In addition to their function and expression on immune cells, certain GPCRs (e.g., GPCR 41 and 43) are also constantly expressed on intestinal epithelial cells, where they sense bacterial-derived metabolites. In recent years it became more and more clear that GPCRs represent a crucial point of contact between the vertebrate host and commensal bacteria in the gut and, consequently, act as key players in the maintenance of gut homeostasis. Their central role in the recognition of both host- and microbiota-derived metabolites was recently reviewed in Husted et al. (165) and the list of GPCR-metabolite interactions is constantly expanding [e.g., (166, 167)]. Interestingly, commensal bacteria produce GPCR ligands that mimic human signaling molecules: Human microbiome-encoded N-acyl amides are structurally similar to endogenous GPCR-active lipids and can activate GPCRs that regulate gastrointestinal tract physiology (168). Moreover, GPCRs on entero-endocrine cells sense microbial metabolites such as SCFAs and seem to mediate the effect of the microbiota on the secretion of hormones and thus on several aspects of host physiology, including behavior [reviewed in (169)].

Thus, in human and mouse, it is well-established that GPCRs detect microbial-derived signals and that the microbiota can affect host physiology through GPCRs.

### Invertebrates

Evidence of the involvement of GPCRs in invertebrate immunity mainly comes from the two model organisms *C. elegans* and *D. melanogaster* [reviewed in (170)]. As an example, in *C. elegans*, the GPCR DCAR-1 was identified as an epidermal DAMP receptor. DCAR-1 responds to the endogenous ligand hydroxyphenyllactic acid, a tyrosine derivate that accumulates in the epidermis upon wounding or fungal infection (45). A Gα protein-PLCβ-DAG-PKC pathway, which then converts onto a p38 MAPK signaling cascades and a STAT-like transcription factor, acts downstream of DCAR-1 to activate the expression of AMPs (44, 45, 171, 172). Moreover, several *C. elegans* GPCRs and GPCR signaling were implicated in the regulation of pathogen avoidance behavior and the expression of intestinal immune defense genes [e.g., (46, 173–178)]. Two *Pseudomonas aeruginosa* secondary metabolites, phenazine-1-carboxamide and pyochelin, activate GPCR signaling in chemosensory neurons, which in turns promotes pathogen avoidance behavior (177). *C. elegans* GPCRs thus present a potential link between the nervous system and immune defenses, it is however as yet unclear if they directly respond to microbial-derived stimuli or to endogenous ligands.

In *D. melanogaster*, GPCRs play a potential role in the activation of dual oxidase (DUOX) enzymes that produce reactive oxygen species, which act as important antimicrobial effectors for the control of pathogens (179). Moreover, the community of commensal bacteria in the gut of DUOX knockdown flies is modified (180). However, DUOX-dependent gut immunity is mainly triggered by opportunistic pathogens, but not by commensal bacteria (63) and DUOX may thus play a role in the discrimination between symbionts and pathogens (181). Genetic and biochemical analyses revealed that DUOX activity and reactive oxygen species production is triggered by the recognition of pathogen-derived uracil (63), and require a Gαq-PLCβ-Ca^2+^ pathway (180). This indicates that DUOX is activated by an upstream GPCR, but the identification of this GPCR remains a challenge for the future.

In other animal models, GPCRs and GPCR signaling components were identified as potential mediators of host-microbe interactions by -omic approaches. For example, GPCR signaling might play a role in the establishment of cnidarian-dinoflagellate endosymbiosis: Transcriptomic and metabolomic analyses of the sea anemone *Aiptasia pallida* response to different endosymbiont *Symbiodinium* species revealed GPCR signaling as one of the major differentially enriched processes (182). In addition, proteomic analysis identified that GPCRs are present in the membranes of symbiosomes, the intracellular vacuoles containing microalgae in a cnidaria-dinoflagellate endosymbiosis (183). Together, studies on different animal models suggest that GPCRs are potential mediators of host-microbe interactions also in invertebrates.

## Perspectives

The metaorganism can only function as entity when host and microbiota efficiently communicate with each other. Here, we presented our current understanding on the role of PRRs and GPCRs in maintaining host-microbiota interactions within the metaorganism throughout different animal groups (Table 1). Accumulating evidence suggests that TLRs, NLRs, CTLDs, and GPCRs are crucial mediators in host-microbiota communication in mammals. However, it remains unclear if this function of these PRRs is conserved throughout the animal kingdom, as is their function in pathogen recognition. Research on PGRPs in diverse animal groups provides strong support of their conserved role in mediating host-microbiota interactions. For mammalian SRs, their interaction with the microbiota seems to be indirect as modulators of host metabolism. In contrast, the work on the sea anemone *Aiptasia* point to a role of SRs in establishment of the symbiosis with *Symbiodinium* and this may be also the case in other animal groups, such as sponges.

Research on animal receptors ranges from comparative genomic studies, differential gene expression or metabolic analysis, biochemical characterization of ligands and receptors and -in a few animal models- functional validation by silencing of target genes and investigation of germ-free vs. conventionally raised animals. In this way, we are advancing our knowledge on the receptors mediating host-microbiota communication but open questions remain. In most cases, the microbiota-derived molecules that the receptors recognize and the signaling pathways that they trigger remain elusive. Moreover, synergies among receptors, which may be key to determine specific responses, are not taken into consideration (184). Understanding these interactions and the downstream signaling upon encounter with different microbes is key to reveal the biological role of PRRs.

Exploring host-microbiota interactions in less complex invertebrate organisms may help to decipher the molecular language used in host-microbiota communication (185). The two most intensively studied invertebrate model organisms are the fruit fly *D. melanogaster* and the nematode *C. elegans*. *D. melanogaster*-gut microbiome research started several years ago with the focus on the effect of gut microbiome on host nutrition [reviewed in (186)], as well as other host functions such as larval growth (187, 188), mate selection (189), and behavior (190, 191). *C. elegans* is only beginning to emerge as model system for metaorganism research (192), but several recent studies have already strengthen the big potential of *C. elegans* to study molecular mechanisms underlying host-microbiota interactions (193–195), including microbiota-mediated protection against pathogens (196). *C. elegans* and *D. melanogaster* both offer experimental advantages for e.g., using large scale genetic screens as tools for receptor identification in host-microbiota interactions, as they combine genetic amenability, low cost, and undemanding culture conditions. They provide the ability to screen knockout mutants or animals, in which genes are individually knockdown by RNAi, for the effect of gene inactivation on microbiota-dependent phenotypes (such as the above mentioned larval growth or microbiota-mediated protection against pathogen infection). These forward genetic screens can be unbiased whole genome screens and may thus offer the potential to discover novel receptors, or targeted on a certain known receptor group, such as GPCRs.

Obviously, not all invertebrates are genetically tractable, have a simple microbiota, and culturable symbionts. However, the study of diverse animal hosts has the potential to bring completely new insights into animal-microbe interactions (197, 198). The early branching metazoan *Hydra* harbors a relatively simple microbiome and offers functional tractability of host and bacteria, in addition to the accessibility of a germfree model. The *Hydra* system helped in deciphering the role of the immune system and the nervous system in shaping the microbiome (199–201) and opening new venues of research, like the role of quorum quenching in host-symbiont interactions (202). The sea anemone *Aiptasia* is arising as a model underlying molecular processes in cnidarian-dinoflagellate symbiosis, which promises to set the basis for understanding coral bleaching (182, 203, 204). Finally, the one-on-one symbiosis between the Hawaiian bobtail squid *E. scolopes* and the bioluminescent bacterium *V. fischeri* has served as beautiful model for studying symbiont colonization and the genetic basis of host-microbe “conversations.” Discoveries such as the release of LPS and PGN signals by *V. fischeri* that trigger the maturation of the host light organ (13) were not only relevant to understanding this specialized and intimate symbiosis, but also broadened our general perspective on host-microbe interactions, changing the focus from pathogenesis to beneficial symbiosis. In this sense, the Vibrio-squid system has been key in fostering the field of metaorganism research.

The study of various invertebrate hosts thus expands the evolutionary view on host-microbiota interactions and provides a high diversity of contexts to interrogate the underlying principles and mechanisms of the metaorganism. For example, as early-diverging animals, sponges offer a window into fundamental processes that have allowed animals to evolve in a microbial world. Despite current low tractability, the diversity of this group allows us to interrogate host-microbiota interactions by comparing different groups; for example the effect of harboring a high dense vs. low dense microbial communities on host immune strategies (21). And even in these complex holobionts, surprising mechanisms of interkingdom interactions have been revealed, like that of “ankyphages”: phage-derived proteins which facilitate the persistence of their host bacteria within the animal host (205). Growing genomic information combined with the amenability of the system to lab manipulation are also bringing echinoderm larva as a promising model for host-microbiota interactions (206, 207). In other invertebrates, metaorganism research is directly link to economic activities. For example, the causes of mortalities in the Pacific oyster *Crassostrea gigas* can only be explained from a holistic approach that addresses immunity-microbe-environment interactions (208). The development of sequencing, imaging, modeling, and genome editing technologies is opening up the mechanisms involved in *a priori* more cryptic animal-microbe interactions.

The study of invertebrate-microbiota interactions broadens the metaorganism concept and provides the wide lens and evolutionary perspective that is required to disentangle core mechanisms of host-microbe interaction as well as the underlying regulatory principles.

## Author Contributions

KD and LP conceived and wrote the manuscript. Both authors contributed to the article and approved the submitted version.

## Conflict of Interest

The authors declare that the research was conducted in the absence of any commercial or financial relationships that could be construed as a potential conflict of interest.
